# A One-step strategy to target essential factors with auxin-inducible degron system in mouse embryonic stem cells

**DOI:** 10.3389/fcell.2022.964119

**Published:** 2022-08-08

**Authors:** Jingsheng Li, Chunhong Dai, Wenyan Xie, Heyao Zhang, Xin Huang, Constantinos Chronis, Ying Ye, Wensheng Zhang

**Affiliations:** ^1^ Cam-Su Genomic Resource Center, Medical College of Soochow University, Suzhou, China; ^2^ Department of Computational Biology St. Jude Children’s Research Hospital, Memphis, TN, United States; ^3^ Department of Biochemistry and Molecular Genetics University of Illinois at Chicago, Chicago, IL, United States; ^4^ Department of Physiology School of Basic Medical Sciences Binzhou Medical University, Yantai, China

**Keywords:** pluripotency, embryonic stem cells, chromatin modifiers, auxin-inducible degron, differentiation

## Abstract

The self-renewal and pluripotency of embryonic stem cells (ESCs) are conferred by networks including transcription factors and histone modifiers. The Auxin-inducible degron (AID) system can rapidly and reversibly degrade its target proteins and is becoming a powerful tool to explore novel function of key pluripotent and histone modifier genes in ESCs. However, the low biallelic tagging efficiency and a basal degradation level of the current AID systems deem it unsuitable to target key pluripotent genes with tightly controlled expression levels. Here, we develop a one-step strategy to successfully target and repress the endogenous pluripotent genes in mouse ESCs and replace their expression with AID fused transgenes. Therefore, this work provides an efficient way for employing the AID system to uncover novel function of essential pluripotent and chromatin modifier genes in ESCs.

## Introduction

The conventional method of studying protein function is based on gene knockout or silencing strategies that targets DNA or RNA in the pre-translational stage (Sauer and Henderson, 1988; Elbashir et al., 2001). The advent of direct protein degradation systems has allowed the uncoupling of protein function from methods that rely on transcriptional silencing. Auxin-inducible degron (AID) technology allows for rapid targeted protein depletion in the presence of the small-molecule auxin (Nishimura et al., 2009)*.* The AID system further requires expression of *Oryza sativa* F-box transport inhibitor response 1 (TIR1) protein which interacts with the native SCF E3 ubiquitin-ligase complex, to target an AID-tagged target protein of interest (Nishimura et al., 2009). Addition of auxin mediates the interaction between the AID-tagged protein and the TIR1-SCF E3 ubiquitin ligase complex, leads to acute target protein degradation by the proteasome. Due to its rapid and reversible (upon auxin removal) degradation nature, the AID system is becoming a widely used method for the functional study of proteins in vertebrate cells, yeast, worms, flies and mice (Holland et al., 2012; Morawska and Ulrich, 2013; Zhang et al., 2015; Baker et al., 2016; Brosh et al., 2016; Perez-Arnaiz et al., 2016; Trost et al., 2016; Wood et al., 2016; Bence et al., 2017; Nora et al., 2017; Friman et al., 2019; Owens et al., 2019; Sybirna et al., 2020).

Compared with traditional genome-editing technologies, the AID system has advantages in terms of specificity, reversibility and time required for *in vivo* protein depletion (Nishimura et al., 2009). It evades the irreversibility of gene knockout strategies, and is not hindered by incomplete silencing and off-target effects (Jackson et al., 2003; Kaelin, 2012). In addition, the AID system provides a powerful research tool for studying highly dynamic biological processes, such as the cell cycle, stem cell differentiation and neural activity (Nora et al., 2017; Ng et al., 2019; Atkins et al., 2020). Unlike other gene silencing strategies, AID-mediated degradation rapid depletion of proteins of interest (POI) within minute or hourly timescales can directly attribute the observed phenotypes to POI function rather than secondary effects arising from the actions of indirect targets (Weiss et al., 2007; Wood et al., 2016). In addition, the reversibility of this method makes performing rescue experiments relatively simple and efficient. Finally, titratable depletion of a POI is useful not only for loss-of-function studies, but also allows the investigation of protein level effects in biological systems (Zhang et al., 2015).

Although auxin-inducible degron technology allows rapid and controlled protein depletion, some pitfalls have been reported (Lambrus et al., 2018; Li et al., 2019; Yesbolatova et al., 2019; Sathyan et al., 2020). For instance, current AID systems can severely degrade target proteins before IAA addition (known as ‘basal degradation’) and in a target-specific manner have exhibited inefficient or incomplete target depletion (Nishimura et al., 2009; Natsume et al., 2016; Daniel et al., 2018; Yesbolatova et al., 2019). To circumvent such issues, Yesbolatova et al. used the *Os*TIR1 antagonist auxinole to suppress the leaky degradation of degron-fused proteins (Yesbolatova et al., 2019). In a similar approach, Li et al. utilized a *At*AFB2-miniIAA7 system that exhibited, faster protein degradation dynamics and lack of basal degradation in the absence of IAA in some types of somatic cells (Li et al., 2019). It is interesting and important to study whether the *At*AFB2-miniIAA7 system could be employed to target critical pluripotency genes in ESCs whose expression levels determine the cell fates.

ESCs are characterized by infinite self-renewal and are pluripotent i.e., have the ability to differentiate into all somatic tissues. Multiple studies have demonstrated the critical role of key pluripotent genes *Oct4*, *Sox2* and *Nanog* for the maintenance of ESCs in a self-renewing state (Ye et al., 2020). Tight expression control of these proteins is essential, as misregulation of *Oct4* and *Nanog* expression levels is shown to result in ESC differentiation (Niwa et al., 2002; Chambers et al., 2003; Chambers and Tomlinson, 2009). Hence, targeting the AID degradation system against proteins whose expression levels are critically linked to cell function and state, requires the use of novel strategies that can bypass limitations associated with basal degradation and low biallelic tagging efficiency.

In this study, we developed a strategy to target the essential pluripotency associated genes *Oct4* and *Nanog* for tightly controlled degradation utilizing a novel AID system implemented in mESC. Our strategy involves transfection of inducible PiggyBac-Oct4-mAID or Nanog-mAID constructs that can functionally replace the endogenously deleted loci and maintain ESCs in their self-renewing state. Our strategy effectively eliminated the basal AID degradation and is not limited by the need for bi-allelic knock-in on endogenous loci observed in traditional AID system as we opted for AID-fused transgenes. Our study provides a simple and efficient method to use the AID system for the functional study of critical genes in ESCs and other types of cells in which expression level is critical for cell survival or differentiation.

## Materials and methods

### Cell culture and differentiation

Mouse ESCs were cultured as previously described (Zhang et al., 2019). Briefly, mESCs were cultured on gelatin-coated Petri dishes with ES medium (DMEM supplemented with 10% fetal bovine serum, 2 mM l-glutamine, 50 mg/ml penicillin, 80 mg/ml streptomycin, 0.1 mM 2-mercaptoethanol (Sigma), and 10^3^ units/mL of leukemia inhibitory factor (LIF; Millipore) at 37 °C and 5% CO_2_. Mouse ESCs were dissociated with 0.05% trypsin in EDTA (Invitrogen).

To induce the degradation of AID tagged proteins, 500 μM of indole-3-acetic acid (IAA, Sigma) was added to cells for ∼24 h or the indicated time.

ESCs dissociated with 0.05% trypsin were centrifuged and re-suspended in ES medium without LIF. 6–8x10^5^ ESCs were then plated in low attachment petri dishes and incubated at 37 °C with 5% CO_2_ to induce the formation of EBs. EBs collection occurred at indicated days for qPCR analysis.

### Plasmid constructions

The synthesized degron tag (mAID or miniIAA7) and eGFP were cloned into pFNF plasmid to generate pL-mAID-eGFP-NeoR and pL-miniIAA7-eGFP-NeoR vectors. Homology arms of targeting vectors were amplified by PCR using mouse genomic DNA as template, and assembled to both sides of mAID-eGFP-NeoR or miniIAA7-eGFP-NeoR cassette by ClonExpress MultiS one-step kit (Vazyme) to generate mAID and miniIAA7 donor vectors. PCR primers and the length of the homology arms are listed in Supplementary Table S1.

SgRNAs were designed by Benchling website (https://www.benchling.com/crispr/) and their sequences are listed in Supplementary Table S1. To generate sgRNA plasmids, PCR amplicons from pUC57 plasmid were ligated to Bsa1 digested pGL3-U6-2sgRNA-CCDB-EF1α-BSD vector.

To generate Rosa26-*At*AFB2 targeting vector, the *At*AFB2-mCherry-weakNLS cassette was amplified by PCR from the pSH-EFIRES-P-*At*AFB2-mCherry-weakNLS plasmid (Addgene #129717) and cloned into pFNF by One-step kit (ClonExpress MultiS one-step kit, Vazyme). The homology arms of *Rosa26* were inserted into the two ends of the *At*AFB2-mCherry-weakNLS cassette to generate the pL-*At*AFB2-mCherry vector.

To generate Oct4-mAID and Nanog-mAID expression vectors, the synthesized mAID was ligated with *Oct4*, or *Nanog* cDNA by overlapping PCR amplification. Then the *Oct4*-mAID and *Nanog*-mAID cassettes were cloned into the pPB-CAG-IRES-Hygro and pPB-CAG-IRES-Zeocin vectors by One-step kit, respectively.

### Generation of AID tagged cell lines

To generate Rosa26-AtAFB2 cells, mESCs cells were transfected with the Rosa26-AtAFB2 targeting vector, pCas9 plasmid (Addgene #41815) and the corresponding sgRNA plasmid using Lipofectamine 3,000 (Invitrogen). After selection with 10 μg/ml blasticidin (BSD) (Invitrogen), colonies were picked up for genotyping.

To generate the cell lines with endogenous genes tagged by miniIAA7 or mAID, pCas9, sgRNA and targeting plasmids were transfected into Rosa26-AtAFB2 or Rosa26-OsTIR1 cells. Colonies were picked up for genotyping after cultured in the selection medium with 250 μg/ml G418 (Sangon Biotech).

To generate ZHBTc4:Oct4-AID cell lines, pPB-CAG-Oct4-mAID-IRES-Hygro, pPB-CAG-OsTIR1-T2A-BSD and pPBase plasmids were transfected into ZHBTc4 cells and cultured in the presence of 1 μg/ml doxycycline to silence the Tet-OFF-Oct4 transgene. Colonies were picked up for genotyping analysis after cultured in the selection medium with 100 μg/ml hygromycin (Sigma) and 10 μg/ml BSD.

To generate RCNβH:Nanog-AID cells, the plasmids pPB-CAG-Nanog-mAID-IRES-Zeocin, pPB-CAG-OsTIR1-T2A-BSD and pPBase were transfected into RCNβH cells, and cultured in the selection medium in the addition of 75 μg/ml of Zeocin (Invitrogen) and 10 μg/ml of BSD.

To generate WT:Nanog-mAID cells, the plasmids pPB-CAG-Nanog-mAID-IRES-Zeocin, pPB-CAG-OsTIR1-T2A-BSD, pPBase, pGL3-U6-sgNanog-Puro and SpCas9 were transfected into E14 cells. After cultured in the selection medium in the addition of 75 μg/ml of Zeocin (Invitrogen) and 1 μg/ml of Puromycin (Sigma), colonies were picked up for genotyping.

For the generation of WT:Oct6-mAID cells, the plasmids pPB-CAG-Oct6-mAID-IRES-Hygro, pPBase, pGL3-U6-sgOct6-Puro and SpCas9 were transfected into Rosa26-OsTIR1 cells. Colonies were picked up for genotyping after cultured in the selection medium with 100 μg/ml Hygromycin (Sigma) and 1 μg/ml of Puromycin (Sigma).

To induce the degradation of miniIAA7 and mAID tagged proteins, 500 μM of IAA was added to cells.

### Genotyping by PCR analysis

Genotyping was carried out by PCR analysis with Taq Master DNA polymerase (Vazyme) with primers listed in Supplementary Table S2. Thermal cycling was performed in Veriti 96-Well PCR instrument (Applied Biosystems) following the protocol of initial denaturation at 95°C for 3 min, 30 cycles of 95°C for 30 s, 55°C for 30 s, 72°C for 1kb/1 min, and ended by the final elongation at 72°C for 5 min.

### Western blot analysis

Western Blots were carried following the protocol as previously described (Zhang et al., 2019). The antibody information was provided in Supplementary Table S3.

### Quantitative RT-PCR

Total RNA was isolated with FastPure Cell/Tissue Total RNA Isolation Kit V2 (Vazyme). cDNA was synthesized with HiScript II Q RT SuperMix (Vazyme). Real-time PCR was performed with Taq Pro universal SYBR qPCR Master Mix (Vazyme). Quantitative RT-PCR was performed in ViiA7 real-time quantitative PCR instrument (Applied Biosystems) following the protocol of initial denaturation at 95°C for 30 s, 40 cycles of 95°C for 10 s, 60°C for 40 s. Gene expression was determined relative to *Gapdh* transcript levels. Standard deviation was calculated from PCR triplicates. Error bars give the SD of three technical qPCR replicates from a representative experiment. qPCR primers were listed in Supplementary Table S4.

## Results

### Targeting *Oct4* with *At*AFB2-miniIAA7 system in mESCs

**FIGURE 1:**
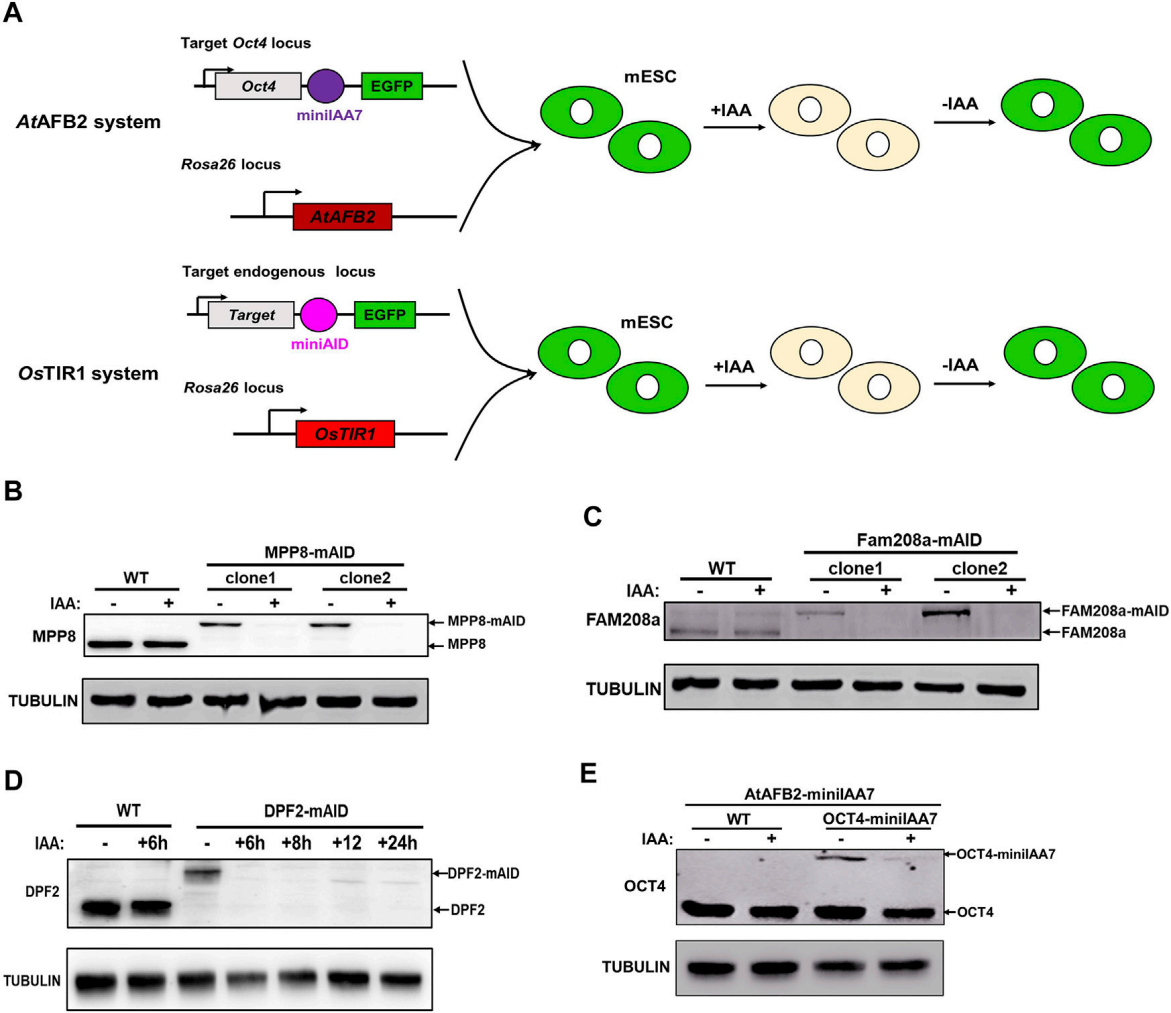
Targeting *Oct4*, *Mpp8*, *Fam208a* and *Dpf2* genes with *At*AFB2-miniIAA7 and *Os*TIR1-miniAID systems in mESCs. **(A)** Experimental scheme to generate Rosa26-*At*AFB2:Oct4-miniIAA7-eGFP and Rosa26-*Os*TIR1:Target gene-miniAID-eGFP ESCs; Immunoblot detection of MPP8-mAID **(B)**, Fam208a-mAID **(C)** and DPF2-mAID **(D)** protein expression. Treatment with 500 µM of IAA for about 24 h degraded the mAID fused proteins. **(E)** Western blotting analysis showed the degradation of Oct4-miniIAA7 protein in the presence of IAA inducer. The Rosa26-*At*AFB2:Oct4-miniIAA7-eGFP cells and WT parental cells were treated with DMSO and 500 µM of IAA respectively for 24 h before Western blot with anti-OCT4 and anti-TUBULIN antibodies.

Compared with the *Os*TIR1 system, the *At*AFB2-miniIAA7 system has higher target protein degradation efficiency and does not exhibit basal degradation in the absence of auxin (Li et al., 2019). Because of these advantages, we utilized the *At*AFB2-miniIAA7 system to target OCT4 protein in mESCs. A parental cell line stably expressing *At*AFB2 was generated by introducing a cassette encoding *At*AFB2 into the *Rosa26* locus with CRISPR/Cas9 technology (Figure 1A). Subsequently a pL-*Oct4*-miniIAA7-eGFP-neo donor plasmid was employed to target miniIAA7-eGFP cassette into the *Oct4* locus upstream of its stop codon. After selection, 144 clones were isolated and genotyped by PCR analysis. 61 clones represented heterozygous insertion (about 42.4%) but no biallelic knock-in clones could be detected (Table 1). We speculate that the low targeting efficiency and lack of biallelic clone is the result of ESC sensitivity to the *Oct4* expression level (Niwa et al., 2000; Niwa et al., 2002) that might be attributed to an observable basal degradation of the OCT4-miniIAA7. Some other factors may also lead to the failure to obtain homogenous AID tagged *Oct4* clones, such as the constitutive expression of *At*AFB2, lower expression level of AID fused OCT4 protein and the poor efficiency of gRNAs employed to target AID to *Oct4* locus et al.

**TABLE 1:** Summary of gene targeting efficiency using AID systems.

Targeted gene	No. of clones	No. of hetero- insertion	Efficiency of hetero-insertion	No. of homo- insertion	Efficiency of homo-insertion
Oct4 (*At*AFB2)	144	61	42.4%	0	0%
Dpf2 (*Os*TIR1)	190	124	65.3%	9	4.74%
Mpp8 (*Os*TIR1)	188	108	57.4%	7	3.72%
Fam208a (*Os*TIR1)	93	52	55.9%	3	3.23%

As a subunit of BAF chromatin remodeling complex, Dpf2 controls the differentiation of mESCs via regulating Tbx3 expression (Zhang et al., 2019). Human silencing hub (HUSH) complex, which consists of MPP8, FAM208a and periphilin, regulates deposition of the epigenetic mark H3K9me3. Recently, MPP8 was reported to be essential to maintain the ground-state of mESCs (Muller et al., 2021). To demonstrate the feasibility of use of the *Os*TIR1-AID systems with ESC biology, we targeted for degradation three chromatin modifier genes, *Dpf2*, *Mpp8* and *Fam208a*. Biallelic tagged clones were identified for all the genes with efficiency ranging from 3.33 to 4.72% (Table 1). The concentration of 500 μM of IAA inducer was used in the whole study as it did not affect the expression of major pluripotency and typical lineage marker genes, the ability of colony formation and cell proliferation of mESCs (Supplementary Figure S1). Western blot verified the efficient degradation of AID tagged MPP8, FAM208a and DPF2 upon IAA inducer treatment (Figures 1B–D) demonstrating the utility of this system for targeting non-pluripotency related genes. Similar to *Dpf2*, *Mpp8* and *Fam208a,* OCT4-miniIAA7 protein can also be degraded in heterozygous cells upon the treatment with 500 μM of IAA for 24 h (Figure 1E). However, the protein level of OCT4-miniIAA7 allele was significantly lower than non-targeted wild type OCT4 allele in heterozygous cells (Figure 1E), indicating the occurrence of basal degradation of OCT4 protein in contrast to the reported lack of basal degradation for *At*AFB2-miniIAA7 systems (Li et al., 2019). These results suggest that additional targeting strategies would need to be considered for the successful implementation of the AID-degradation system against essential pluripotent genes in mESCs.

### Generation of ZHBTc4:Oct4-AID and RCNβH:Nanog-AID ESCs

**FIGURE 2:**
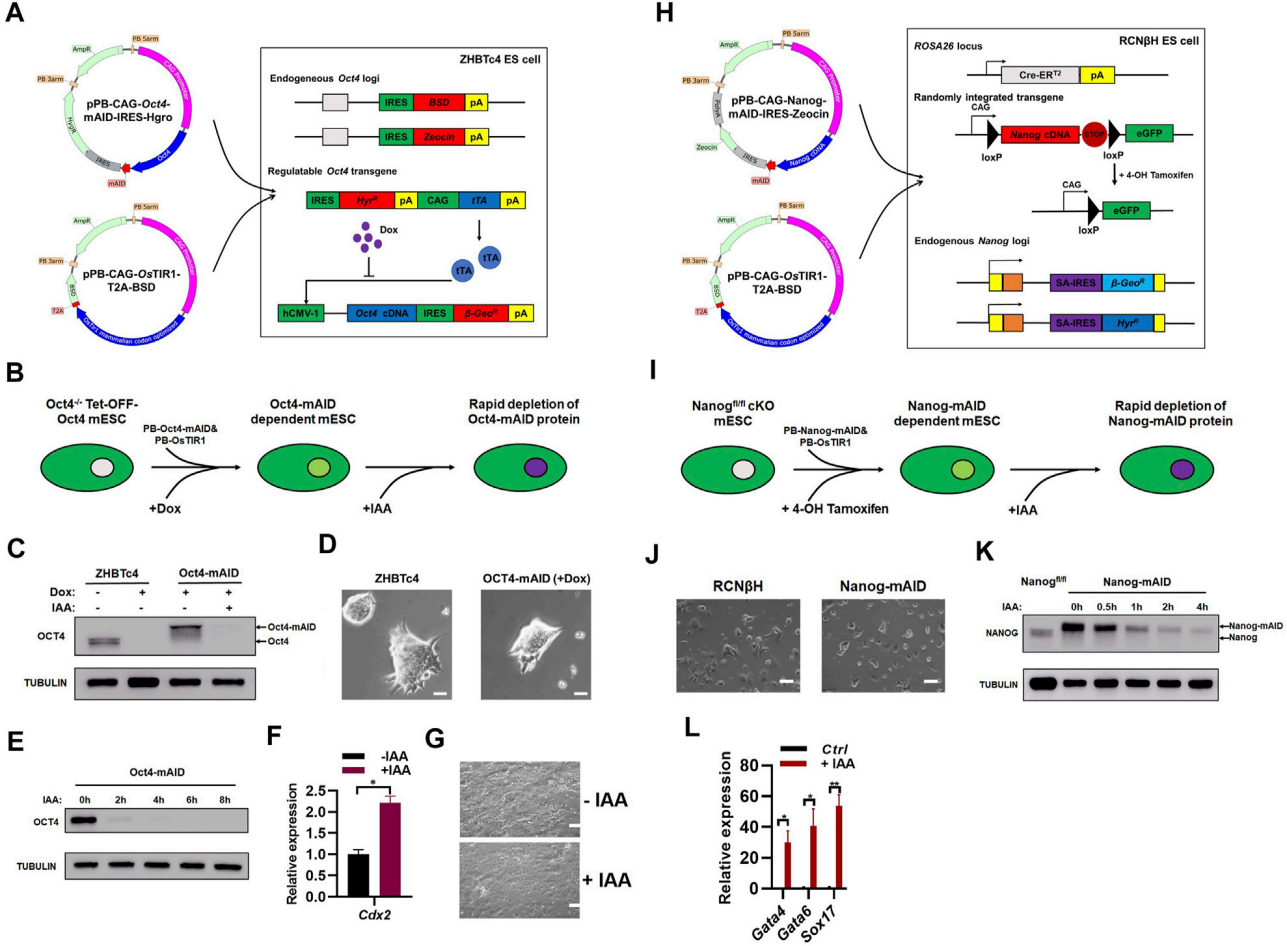
Generation of OCT4 and NANOG proteins with a novel AID system. **(A)** Generation of ZHBTc4:Oct4-AID cell line. The pPB-CAG-Oct4-mAID-IRES-Hygro was produced by fusing mAID to the C-terminus of *Oct4* cDNA, separated by a 5-amino acid short linker composed of GGSGG). ZHBTc4 ESCs were transfected with pPB-CAG-Oct4-mAID-IRES-Hygro, pPB-CAG-*Os*TIR1-T2A-BSD and pPBase vectors, and selected with hygromycin and BSD. During the selection, doxycycline was added to repress the Tet-OFF-Oct4 transgene. **(B)** Schematic diagram shown the generation of ZHBTc4:Oct4-AID mESCs and auxin induced depletion of OCT4-AID protein. **(C)** Immunoblot detection of OCT4-mAID and OCT4 in ZHBTc4 and ZHBTc4:Oct4-AID ESCs. Treatment with of IAA for 24 h degraded OCT4-mAID fusion proteins in ZHBTc4:Oct4-AID ESCs. **(D)** The typical morphology of ZHBTc4 (left) and ZHBTc4:Oct4-AID (right) ESCs. The ZHBTc4:Oct4-AID cells grown in the presence of 1 μg/ml of doxycycline. Scale - 50 microns. **(E)** Time course experiment to determine the time required for depletion of the OCT4-mAID protein. The ZHBTc4:Oct4-AID ESCs grown in the presence of 1 μg/ml of doxycycline were treated with 500 μM of IAA for the indicated time. The levels of OCT4-mAID protein were analyzed by western blotting using anti-OCT4 antisera. TUBULIN protein was used as a loading control. **(F)** qPCR analysis of transcript levels of *Cdx2* gene in ZHBTc4:Oct4-AID ESCs treated with/without IAA. The error bars indicate three independent biological replicates (mean ± SD), *p < 0.05, **p < 0.001, ***p < 0.0001. **(G)** The typical morphology of ZHBTc4:Oct4-AID ESCs treated with or without IAA for 3 days. Scale - 100 microns. **(H)** Generation of RCNβH:Nanog-AID cell line. To generate pPB-CAG-Nanog-mAID-IRES-Zeocin vector, mAID was fused to the C-terminus of mouse *Nanog* cDNA, separated by a 5-amino acid short linker composed of GGSGG. RCNβH ESCs were transfected with pPB-CAG-Nanog-mAID-IRES-Zeocin, pPB-CAG-*Os*TIR1-T2A-BSD and pPBase, and selected with Zeocin and BSD. During the selection, 4-OHT was added to knock out floxed Nanog in RCNβH cells. **(I)** Schematic diagram showing the generation of RCNβH:Nanog-AID mESCs and auxin induced depletion of NANOG-mAID protein. **(J)** The typical morphology of RCNβH (left) and RCNβH:Nanog-AID (right) ESCs. Scale - 100 microns. **(K)** Immunoblot detection of NANOG-mAID and NANOG in RCNβH: Nanog-AID ESCs and NANOG in RCNβH (*Nanog* ^*fl/fl*^) ESCs. Treatment with 500 μM of IAA for the indicated time degraded NANOG-mAID fusion proteins in RCNβH:Nanog-AID ESCs. The levels of NANOG-mAID protein were analyzed by western blotting using anti-NANOG antisera. TUBULIN protein was used as a loading control. **(L)** qPCR analysis of transcript levels of *Gata4*, *Gata6* and *Sox17* genes in 3 days’ embryoid bodies induced from RCNβH:Nanog-AID ESCs treated with/without IAA. The error bars indicate three independent biological replicates (mean ± SD), *p < 0.05, **p < 0.001, ***p < 0.0001.

Considering the sensitivity of ESC maintenance to the *Oct4* expression level (Niwa et al., 2000; Niwa et al., 2002), the basal degradation of OCT4-miniIAA7 protein in the *At*AFB2-miniIAA7 system might be the main obstacle to generate homogenous miniIAA7 tagged *Oct4* ESCs. Therefore, a strategy to maintain ESCs by overexpressing exogenous OCT4-mAID fusion protein in *Oct4* KO ESCs could potentially allow the generation of ZHBTc4:Oct4-mAID ESC cell lines. Niwa et al. established the Tetoff-*Oct4* cell line (ZHBTc4) through genetic manipulation of mESCs (Niwa et al., 2000; Niwa et al., 2002). In this system, the addition of 1 μg/ml of doxycycline is sufficient to completely repress *Oct4* expression (Niwa et al., 2002). To generate ZHBTc4:Oct4-mAID mESC cell lines (hereinafter referred to as ZHBTc4:Oct4-AID mESC), PiggyBac *Oct4*-mAID and *Os*TIR1 vectors were transfected to ZHBTc4 cells (Figure 2A). The PiggyBac system ensures insertion of multiple copies of the *Oct4*-mAID transgenes. Importantly, in the continuous presence of doxycycline the generated cell line that remain pluripotent would be through the expression of mAID tagged *Oct4* (Figure 2A).

We first performed auxin-induced degradation experiments in ZHBTc4:Oct4-AID cells (Figure 2B). In the presence of doxycycline, ZHBTc4:Oct4-AID cells only expressed OCT4-mAID fusion protein at a level that was slightly higher than the endogenous OCT4 protein (Figure 2C) (Niwa et al., 2000), which might be due to the stronger activity of CAG promoter than the endogenous *Oct4* promoter. In the presence of doxycycline, the addition of 500 µM of IAA degraded OCT4-mAID fusion protein (Figure 2C; Supplementary Figure S2A). ZHBTc4:Oct4-AID cells in the presence of doxycycline showed similar morphology to the parental ZHBTc4 ESC cell line (Figure 2D), indicating the high expression of OCT4-AID fusion protein is still within the level than can maintain ESC morphology and basic properties of self-renewal. In the presence of doxycycline, addition of 500 µM of IAA was sufficient to effectively degrade the OCT4-mAID fusion protein in ZHBTc4:Oct4-AID cells within a 2-h window of IAA addition (Figure 2E; Supplementary Figure S2B). Consistent to the report by Niwa et al. (Niwa et al., 2005), the degradation of the OCT4-mAID fusion protein in ZHBTc4:Oct4-AID cells (in doxycycline conditions) induced the expression of *Cdx2* and the cells showed morphology of differentiation (Figures 2F,G).

Chambers et al. generated a *N*anog conditional knockout ES cell line (RCNβH) using traditional genetic manipulations (Chambers et al., 2007). Treatment of RCNβH cells with 0.5 μM of tamoxifen (4-OHT) resulted in the loss of *Nanog* expression (Chambers et al., 2007). In a similar manner to the ZHBTc4:Oct4-AID ESC line, Nanog-mAID mESCs (hereinafter referred to as RCNβH:Nanog-AID mESC) were obtained after co-transfection of the PiggyBac Nanog-mAID and *Os*TIR1 vectors into RCNβH cells (Figure 2H). Auxin-induced degradation experiments with RCNβH:Nanog-AID cells were performed in a similar manner to their *Oct4* counterparts (Figures 2B,I). RCNβH:Nanog-AID cells and the RCNβH parent cell line exhibited similar cell morphology (Figure 2J), suggestive of Nanog-mAID construct ability to perform equivalent functions to its endogenous counterpart in ESC maintenance. The floxed *Nanog* alleles were deleted upon the treatment of ESCs with 1 μM of 4-OHT for a 2-day period. Subsequent addition of 500 μM of IAA as short as 2 h could efficiently degrade the NANOG-mAID protein (Figure 2K; Supplementary Figure S2C). Due to the stronger activity of CAG promoter than endogenous *Nanog* promoter, the expression level of NANOG-mAID protein was higher than the endogenous NANOG protein (Figure 2K). Degradation of NANOG-mAID protein induced the expression of endoderm marker genes *Gata4*, *Gata6*, *Sox17* in embryoid bodies allowed to form over a 3-day period (Figure 2L), which is consistent to previous reports of NANOG loss (Chambers et al., 2003; Mitsui et al., 2003).

### One-step generation of WT:Nanog-AID and WT:Oct6-AID ESCs

**FIGURE 3:**
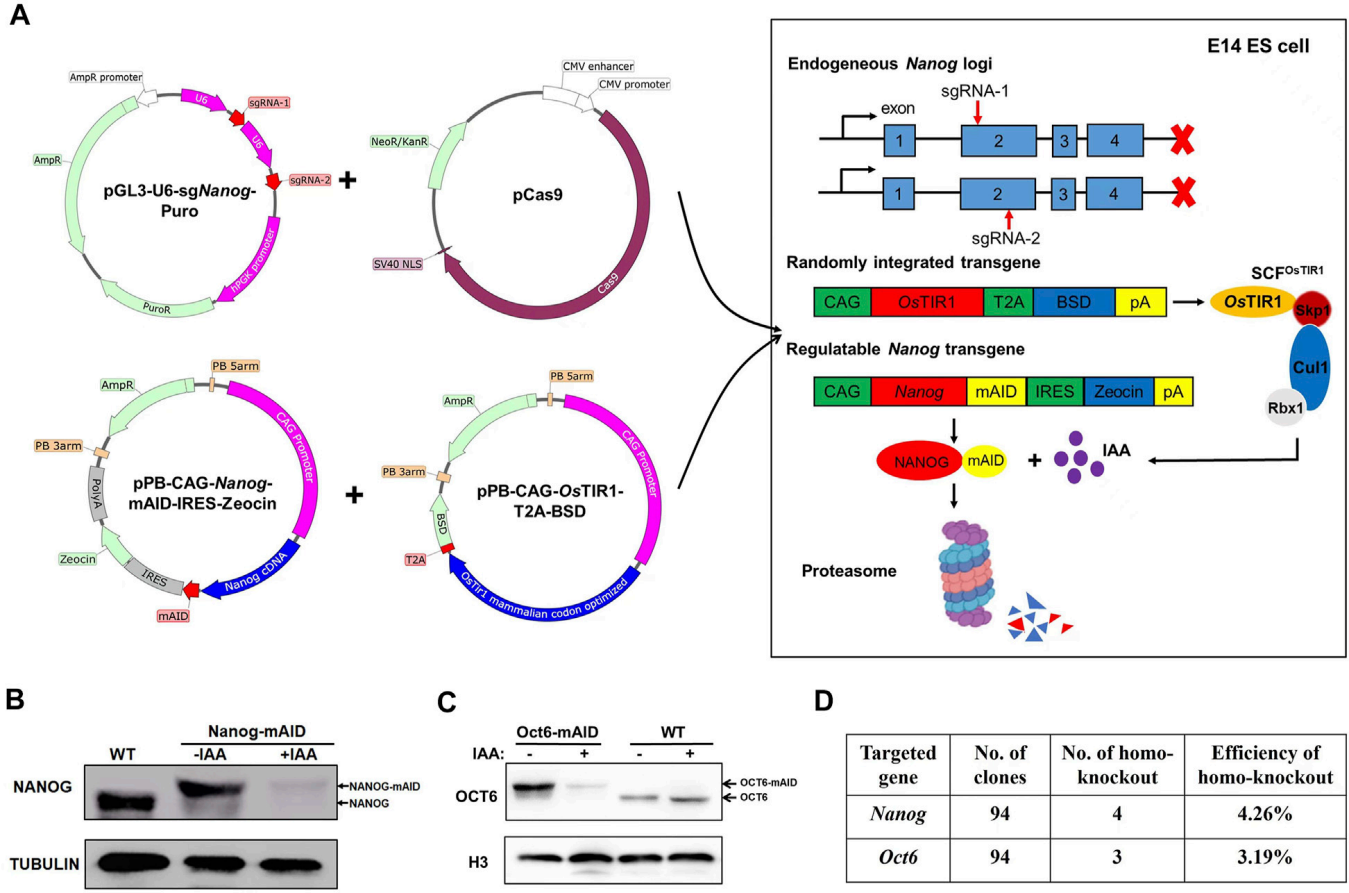
Generation of WT:Nanog-AID and WT:Oct6-AID cell lines in a one-step strategy. **(A)** Schematic diagram shown the generation of WT:Nanog-AID mESCs by a one-step strategy. **(B)** Immunoblot detection of NANOG-mAID and NANOG in WT:Nanog-AID ESCs. The addition of 500 μM of IAA for 24 h degraded NANOG-mAID fusion proteins. **(C)** Immunoblot detection of OCT6-mAID and OCT6 in WT: Oct6-AID ESCs. The addition of 500 μM of IAA for 24 h degraded OCT6-mAID fusion proteins. **(D)** The efficiency to generate WT:Nanog-AID and WT:Oct6-AID ESCs using a one-step strategy.

The generation of ZHBTc4:Oct4-AID and RCNβH:Nanog-AID ESCs is limited by a dependence on the pre-existence of conditional *Oct4* and *Nanog* ESCs lines (Figures 2A,F). Hence, a strategy to knockout an endogenous target gene by CRISPR/Cas9, and replace it with exogenously expressed AID tagged gene utilizing a single-step method would significantly simplify the process and generalize the use of our AID tagged ESC model. To test the effectiveness of such a method, PiggyBac (PB) Nanog-mAID and PB-*Os*TIR1 were transfected with Cas9/gRNA vectors, which were used to inactivate the endogenous *Nanog* gene in mESCs (Figure 3A; Supplementary Figure S3A). Following the above strategy, WT:Nanog-AID mESC clonal lines were obtained following drug selection and genotyping. Addition of IAA efficiently degraded the NANOG-mAID protein in the generated lines exemplifying the feasibility of our approach (Figure 3B; Supplementary Figure S3B). Unfortunately, we failed to generate Oct4-mAID cell line with the one-step strategy. To exemplify the simplicity of our approach, we generated an additional cell line targeting the *Oct6* gene for degradation. WT:Oct6-AID ESC lines were generated using our one-step strategy. Cas9/gRNA plasmids were transfected to inactivate the endogenous Oct6 (Supplementary Figure S3C). The addition of IAA was shown to degrade OCT6-mAID protein (Figure 3C; Figure S3D). The relative efficiencies to obtain WT:Nanog-AID and WT:Oct6-AID cell lines using our one-step strategy were 4.26 and 3.19%, respectively (Figure 3D), a significant improvement over current AID-targeting methods.

Collectively, the above results demonstrate a novel strategy to use AID rapid degradation towards targeted proteins whose expression levels are tightly controlled and whose deregulation leads to loss of cell identity. The system reported here effectively overcomes the basal degradation of AID-targeted proteins and the resulting side-effects, thus providing a simple way to explore the mechanisms by which such target proteins contribute to cellular function.

## Discussion

The AID system ensures specific, fast and reversible degradation of a target protein and allows the investigation of complex biological processes in a fine-tuned manner. The development of CRISPR-Cas9 genome-editing technology has further streamlined the process of endogenous gene tagging in mESCs allowing the functional investigation of proteins of interest (Baker et al., 2016; Natsume et al., 2016).

The key factors for the success of the AID system are the high and stable expression of F-box proteins (*Os*TIR1 and *At*AFB2) and the production of highly integrated homozygous clones (Nishimura et al., 2009; Li et al., 2019). The continuous expression of the auxin-interacting F-box protein is one of the necessary conditions for the proteasome to effectively degrade AID-labeled proteins (Nora et al., 2017). In theory, integration of the *Os*TIR1 in a safe harbor locus can provide sufficient expression levels. This approach was recommended in a majority of protocols where many suitable loci (*H11*, *TIGRE*, *AAVS1*, *ROSA26*) were extensively characterized (Baker et al., 2016; Nora et al., 2017; Pryzhkova et al., 2020). However, the disadvantages of the safe harbor targeting method include the use of multiple targeting steps, low relative expression levels and sensitivity to silencing. For example, the reported expression of the *TIGRE* safe harbor locus is prone to silencing or downregulation after ES cell differentiation (Nora et al., 2017). In our study, *At*AFB2/*Os*TIR1 is integrated into the *Rosa26* locus to generate Rosa26-*At*AFB2 and Rosa26-*Os*TIR1 parental cells (Figure 1A). For both non pluripotent and pluripotent genes studied in this work, the tagged proteins were degraded upon the IAA induction (Figures 1, 2), indicating that *At*AFB2 or *Os*TIR1 expression from the *Rosa26* locus is sufficient for the AID degradation system to function (Natsume et al., 2016; Li et al., 2019) (Table 1). Nevertheless, Yunusova et al. demonstrated that there is no significant difference between transgenic integration on safe harbor loci and random integration for parts of the AID system machinery (Yunusova et al., 2021). Consistent with this result, we employed PiggyBac integration system (PB-*Os*TIR1) to efficiently degrade NANOG-mAID and OCT6-mAID in WT:Nanog-AID and WT:Oct6-AID ESCs, respectively (Figures 3B,C). Random integration of transgenes via a PiggyBac integration mechanism could also be utilized for *Os*TIR1 transgenes insertion and expression. Considering all benefits of employing the PiggyBac integration mechanism we should note that random integration can potentially disrupt essential genes affecting cell survival, proliferation, protein synthesis, cell identity maintenance, etc.

A number of studies have demonstrated that the ubiquitous expression of *Os*TIR1 can lead to "basal level degradation” of some target proteins. Since the observed degradation is not auxin dependent, improved systems strive to address this issue (Li et al., 2019; Sathyan et al., 2019; Yesbolatova et al., 2019). Li et al. reported that the *At*AFB2-miniIAA7 system is superior to the mAID system, with faster degradation dynamics and lack of basal degradation (Li et al., 2019). However in our study, basal degradation of OCT4-miniIAA7 was still observed in Rosa26-*At*AFB2:Oct4-miniIAA7 heterogenous clones (Figure 1E). The observed discrepancy may be gene or cell type dependent, which requires further exploration in future studies. Later, after we have started exploring the strategies in this study, two groups reported a novel system which employed the AID2 system with an *Os*TIR1(F74G) mutant and 5-Ph-IAA ligand, no basal degradation of target proteins were detected in HCT116 cells (Yesbolatova et al., 2020) and AID tagged proteins were degraded with significantly lower concentration of 5-Ph-IAA inducer (Nishimura et al., 2020; Yesbolatova et al., 2020). It would be interesting to study whether AID2 system could eliminate basal degradation of target proteins in ESCs.

In previous works, auxin induced degradation of protein-of-interest was normally achieved via tagging endogenous genes through CRISPR/Cas9 technology (Natsume et al., 2016; Li et al., 2019). CRISPR-associated limitations include aberrant loci deletion, low efficiency of homozygous knock-in and non-characterized effects in gene expression. Such issues can generate ambiguities in phenotypic studies and pose a significant challenge for the generic adoption of CRISPR technology for the generation of AID mediated cell line engineering (Irion et al., 2007; Smith et al., 2008; Sakurai et al., 2010; Nora et al., 2017). Of note, AID targeting of the essential pluripotency genes *Oct4* and *Nanog*, has been challenging due to the fact that pluripotency-related transcription factor expression levels are finely regulated and essential to the maintenance of ESCs (Ye et al., 2020). Here, we developed a strategy that allows ESCs to maintain their pluripotency by expressing OCT4-mAID or NANOG-mAID fusion protein (Figures 2, 3). Recently, Bates et al. generated OCT4-AID ESCs in a similar way and identified novel mechanism of *Oct4* function (Bates et al., 2021). In our method, no basal degradation and leakage defects that can lead to aberrant phenotypic losses were observed. By simply overexpressing AID tagged proteins of interest in an endogenous CRISPR-mediated knock-out setting, we are able to target most genes whose expressions are essential for physiological cell function. With the strategy, WT:Nanog-AID and WT:Oct6-AID cell lines were generated and NANOG-mAID and OCT6-mAID fusion proteins were degraded efficiently while maintaining ESC morphology and function prior to the addition of IAA. However, caution should be taken to interpretate the data generated from such cell lines to ensure that the observed phenotypes are not resulting from the random insertion of the AID tagged cDNA. The employment of CRISPR-mediated knock-out in the one-step method may produce phenotypes unrelated to the function of AID target genes. Generation of multiple clones with distinct integration events can circumvent such issues.

In this study, we demonstrate the limitations of recently reported methods in addressing basal degradation of POIs in mESCs. Therefore, an efficient strategy to generate AID tagged cell lines was developed by replacing endogenous target genes with exogenously expressed AID tagged target genes. Our approach eliminates basal degradation of POIs and avoids the use of biallelic tagging strategies, thus extending the application of AID technology to other types of cells with inefficient knock-in capabilities.

## Data Availability

The original contributions presented in the study are included in the article/Supplementary Material, further inquiries can be directed to the corresponding authors.
