# Supplementary figures and images for: A One-step strategy to target essential factors with auxin-inducible degron system in mouse embryonic stem cells
Source: Front Cell Dev Biol. 2022 Aug 8;10:964119. doi: 10.3389/fcell.2022.964119 (PMC9393215; doi:10.3389/fcell.2022.964119)

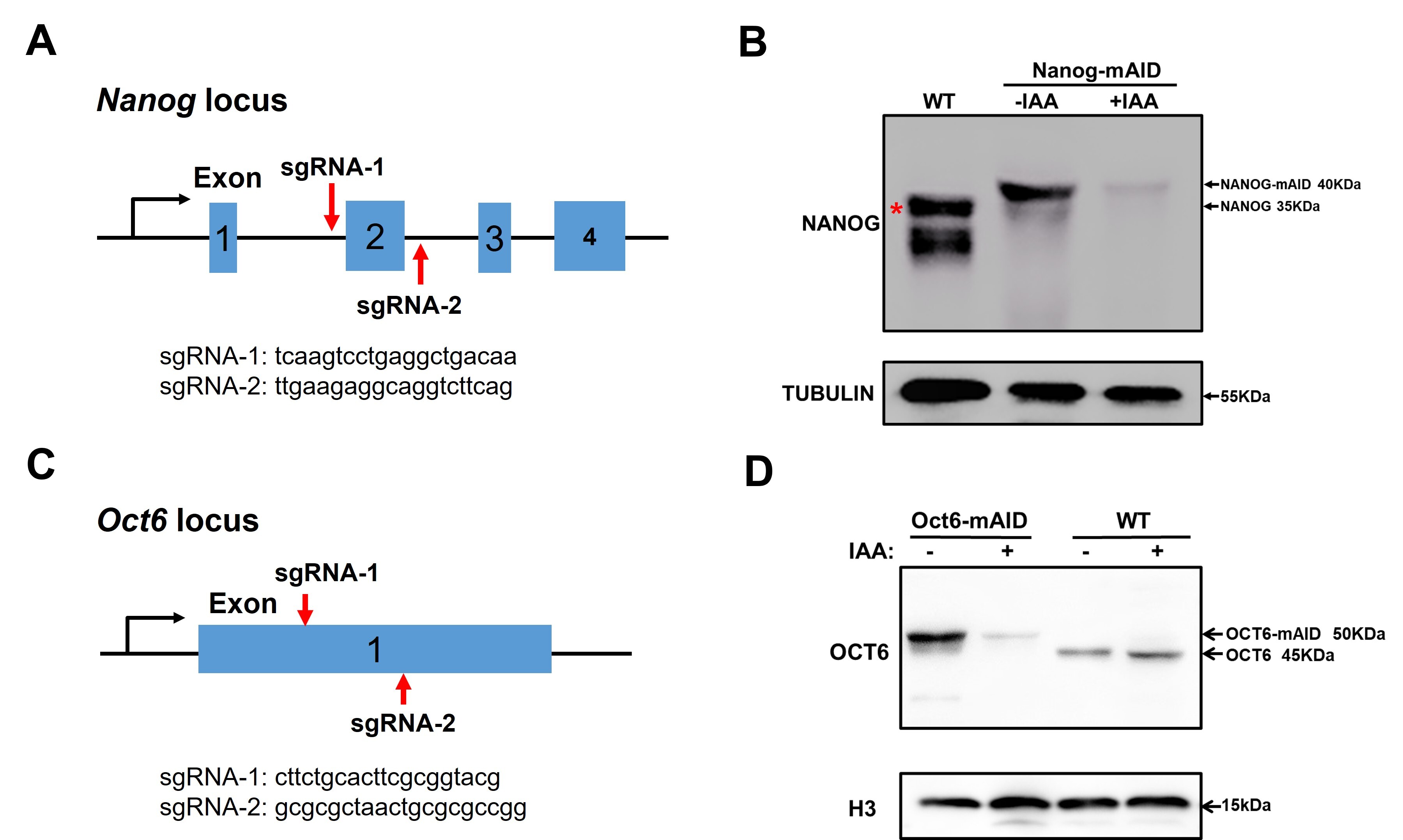

Supplement: Supplementary file 1 [file Image3.JPEG]

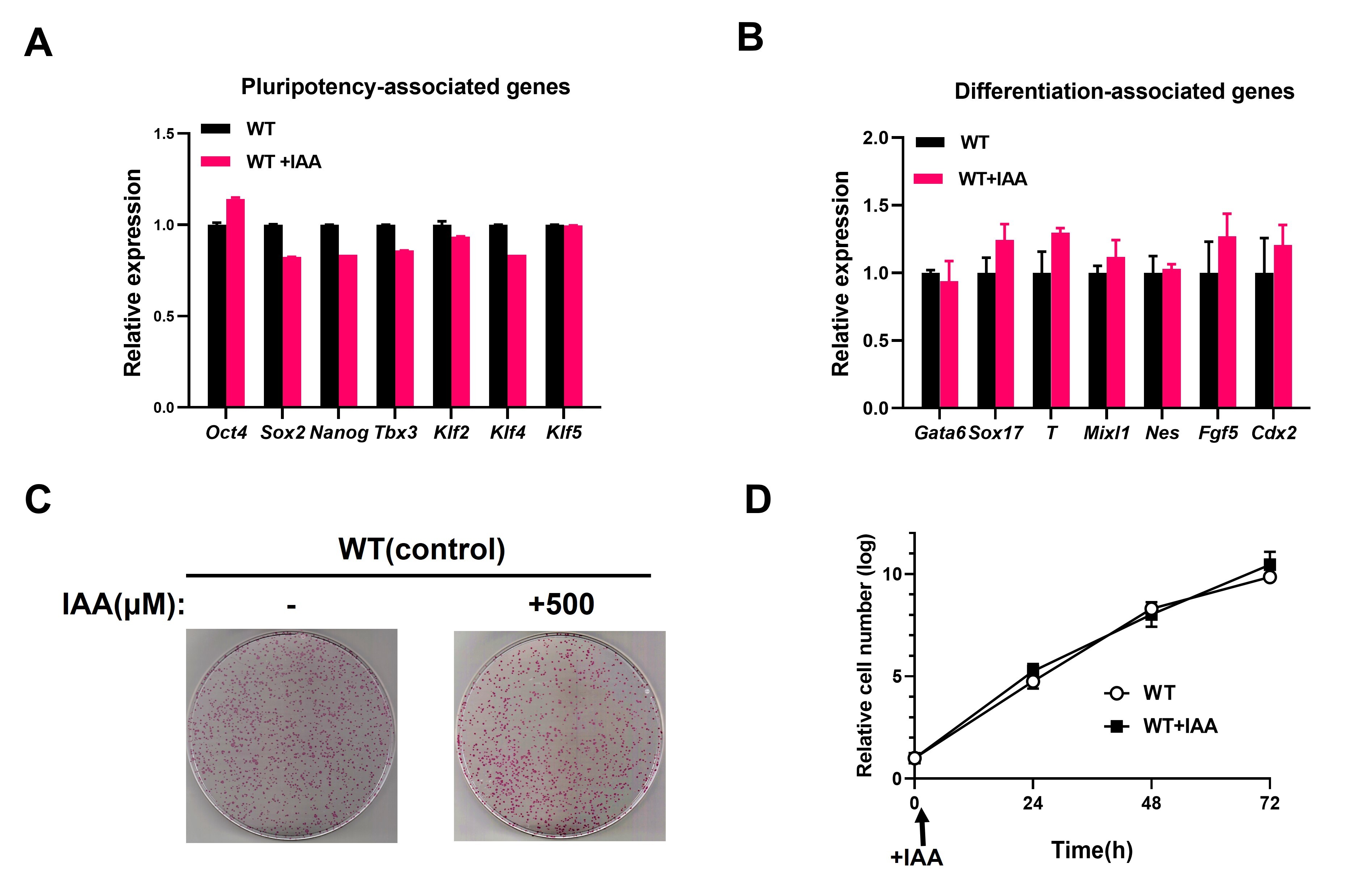

Supplement: Supplementary file 3 [file Image1.JPEG]

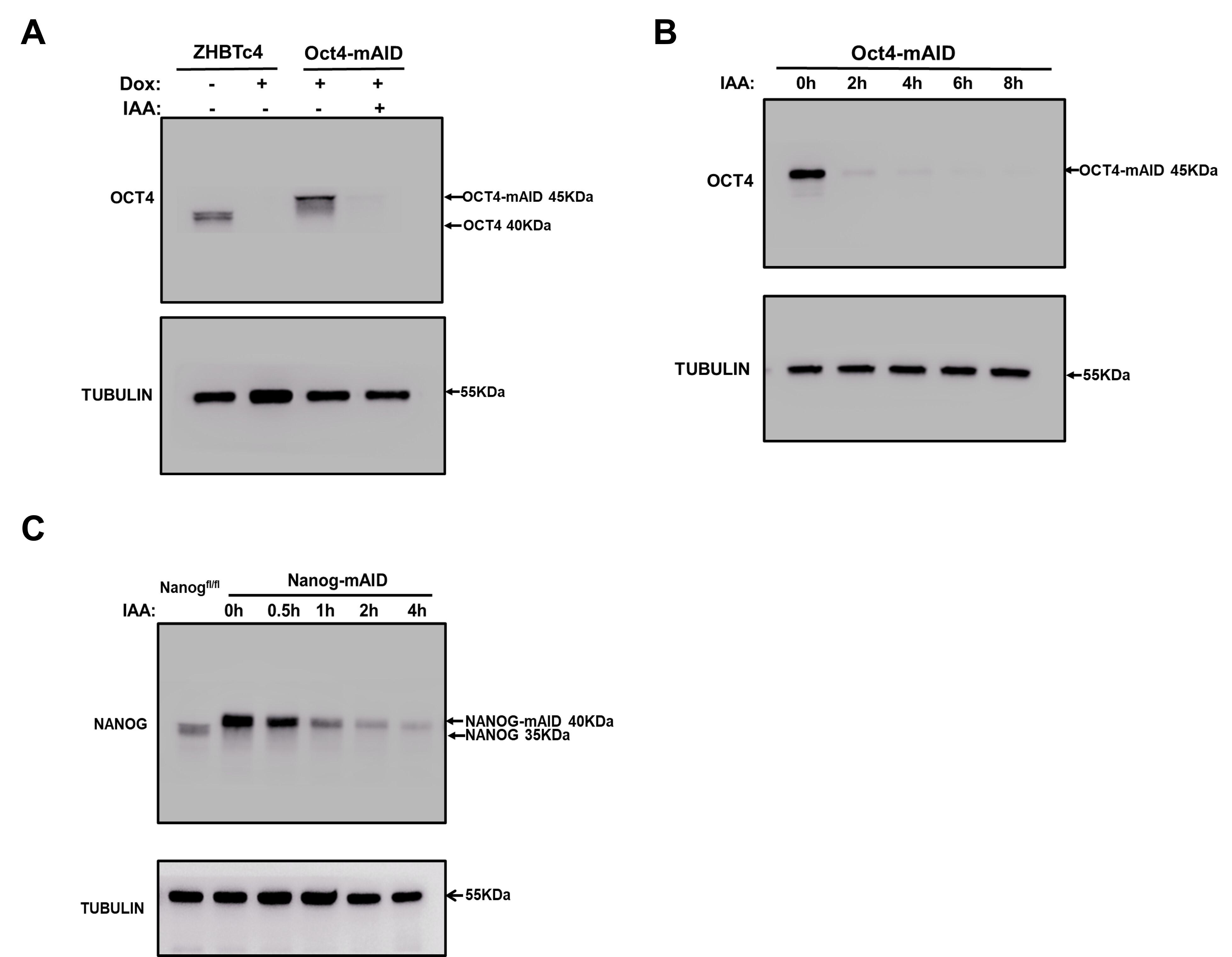

Supplement: Supplementary file 4 [file Image2.JPEG]
