# Supplementary material for: A One-step strategy to target essential factors with auxin-inducible degron system in mouse embryonic stem cells
Source: Front Cell Dev Biol. 2022 Aug 8;10:964119. doi: 10.3389/fcell.2022.964119 (PMC9393215; doi:10.3389/fcell.2022.964119)

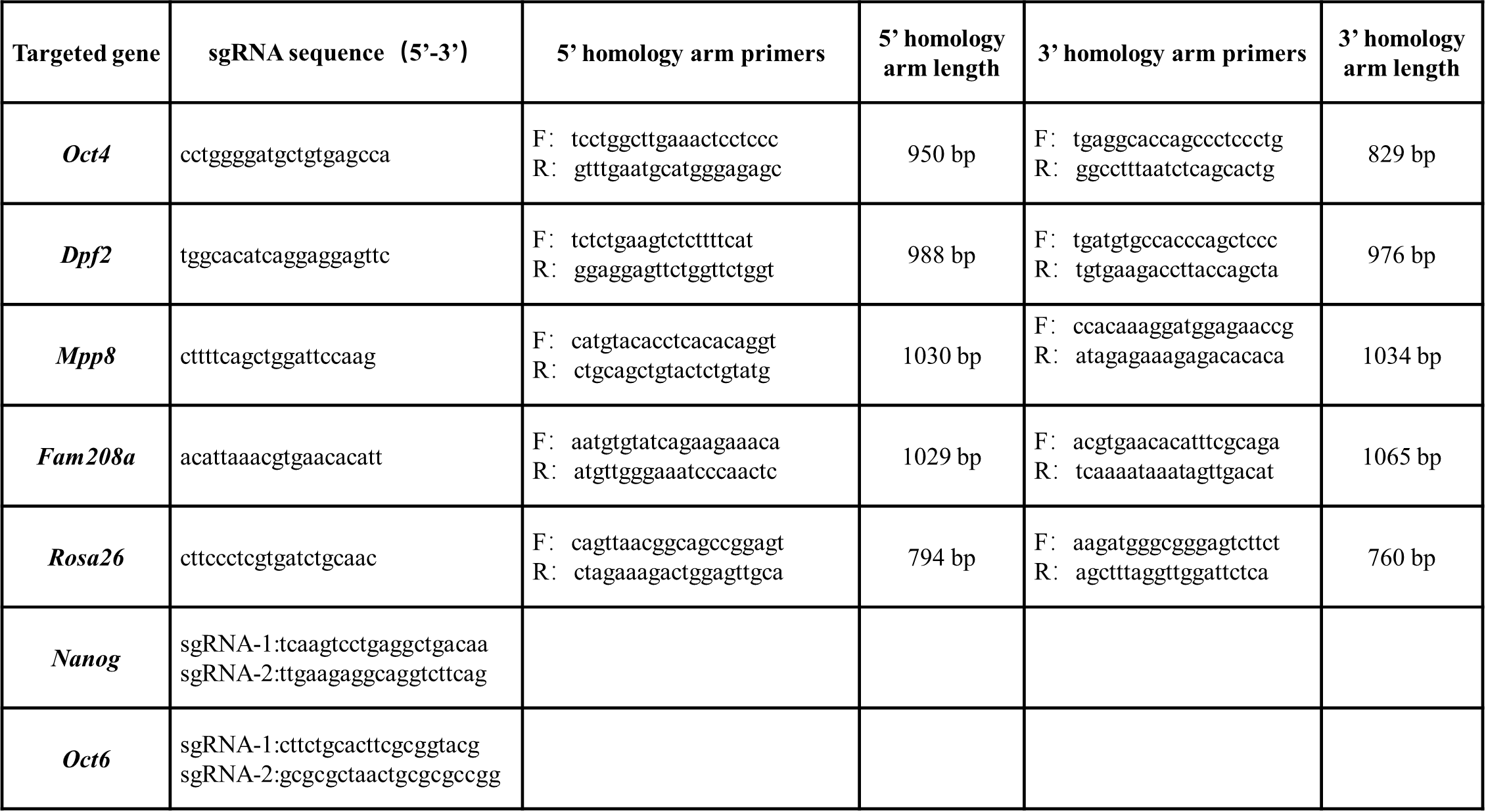
**Table S1.** The sgRNA sequences and PCR primer sequences for homology arm amplification.

**Table S2.** Primers for genotyping analysis.


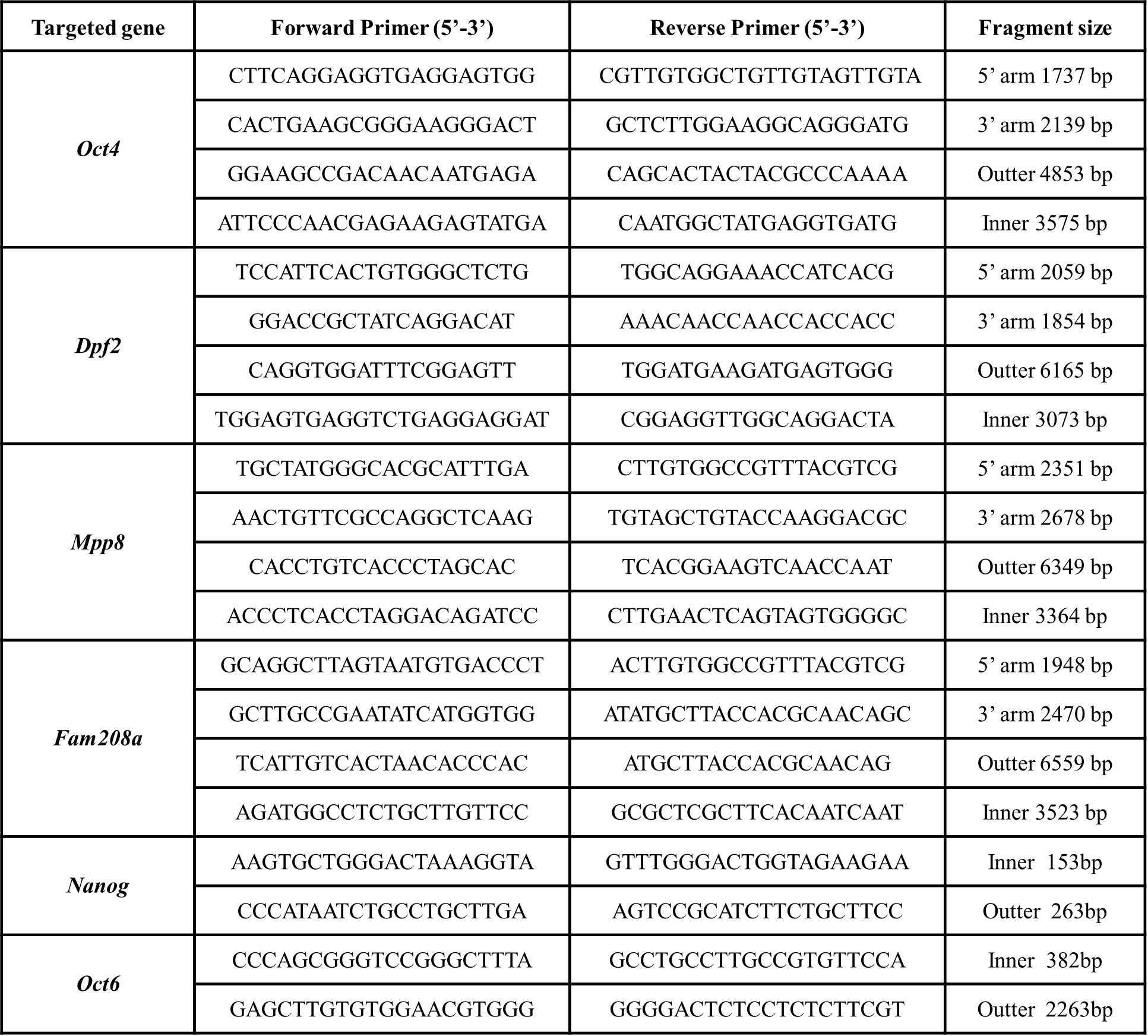


**Table S3.** Antibody information for Western blot analysis.

**
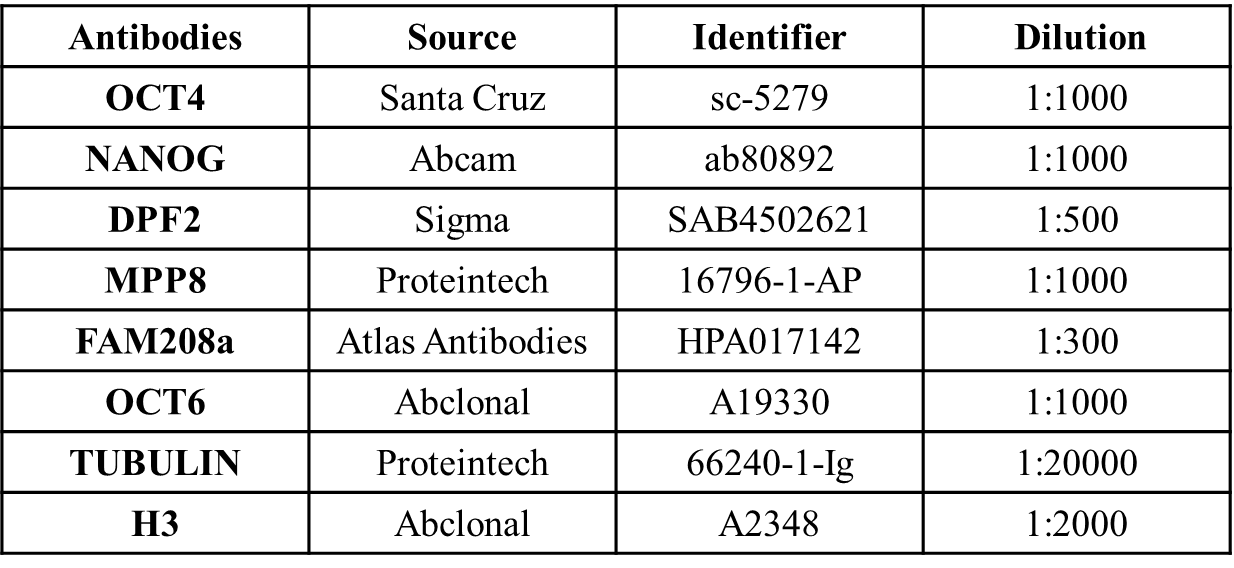
**

**Table S4.** Primers for qPCR.


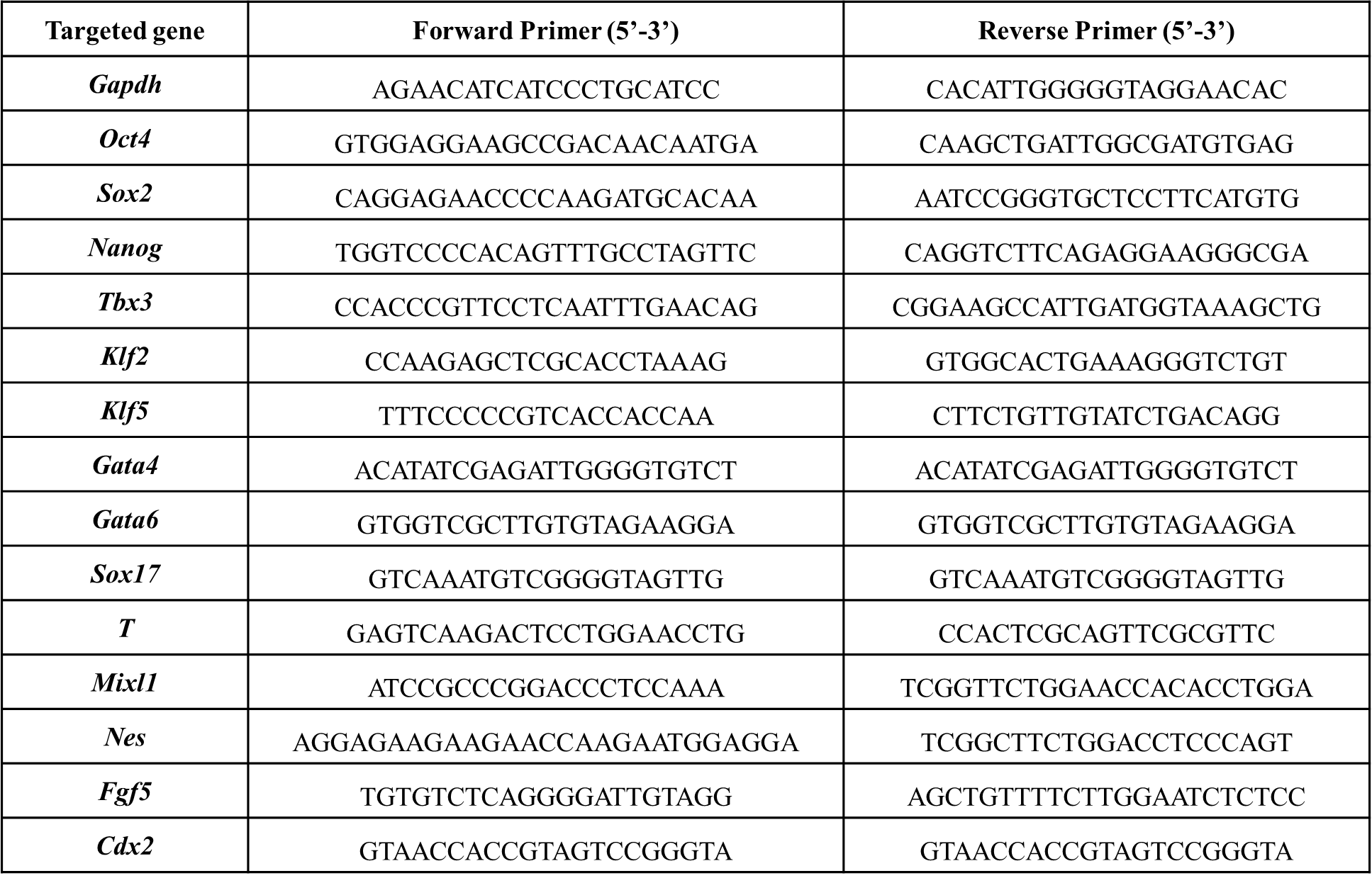

Supplement: Supplementary file 2 [file Table1.DOCX]
